# 

**Published:** 1898-05-14

**Authors:** 


**Notices of Births, Deaths, and Marriages** are inserted in this column at a charge of 2s. 6d. for an announcement not exceeding four lines.

### NOTICE TO CORRESPONDENTS.

All MS., Letters, Books for Review, and other matters intended for the Editor should be addressed The Editor, "The Hospital," The Hospital Building, 28 & 29, Southampton Street, Strand, W.C.

The Editor cannot undertake to return rejected MS., even when accompanied by stamped directed envelope.

**Notice to Advertisers and Subscribers.**

All Advertisements, Orders for Copies of The Hospital, and Business Communications should be addressed to **THE MANAGER, (Not to The Editor),** The Hospital, The Hospital Building, 28 & 29, Southampton Street, Strand, London, W.C.

The Cost of Subscription, post free, is as follows:—Per week, 2½d. per quarter, 2s. 8d.; per half-year, 5s. 3d.; per annum, 10s. 6d. Foreign:—Per annum, 15s. 2d.; payable, in all cases, in advance.

**NOTICE.—Vol.XXIII. of "The Hospital" (October, 1897, to March, 1898), in handsome cloth binding, is now on sale at the Office of this Journal, price 7s. 6d. Cases for binding the Numbers contained in this Volume, 1s. 6d. Index to Vol. XXIII., 2½d., post free.**

**The Monthly Part for May is now ready. Price 6d., by post 8d.**

### BOOKS RECEIVED.

JOHN WRIGHT AND CO.
"Diseases of the Upper Respiratory Tract." By P. Watson Williams, M.D. Lond.

WATTS AND CO.
"Responsible or Irresponsib'e." By Henry Smith, M.D. (Jena).

MARSHALL BROTHERS.
"Health in the Mission Field." By Dr. C. F. Harford Battersby.

FANNIN AND CO.
"Transactions of the Royal Academy of Medicine in Ireland." Vol. XV.

J. AND A. CHURCHILL.
"Chevasse's Advice to a Wife." Revised by Fancourt Barnes, M.D.
"Chevasse's Advice to a Mother." By George Carpenter, M.D. Lond.

ISBISTER AND CO.

"Carlisle Cathedral." By R. S. Ferguson, F.S.A. Illustrated by Alexander Ansted.

"WESTERN MAIL," CARDIFF.
"Handbook of the Workmen's Compensation Act, 1897." By M. Roberts-Jones.

THE SCIENTIFIC PRESS.

"Notes on Pharmacy and Dispensing for Nurses." By C. J. S. Thompson.

"GUARDIAN" OFFICE, EARLESTOWN.
"Supplement to the Annual Report to the Urban Sanitary District of Haydock, 1897." By J. S. Hayward, M.D. Lond., M.O.H.

CASSELL AND CO.
"Ringworm in the Light of Recent Research." By Malcolm Morris.

THE GENERAL MEDICAL COUNCIL.
"The British Pharmacopœia, 1893."

ADAM AND CHARLES BLACK.
"Diseases of the Heart and Aorta." By George William Balfour, M.D., LL.D.

SAMPSON LOW, MARSTON, AND CO.
"A Complete System of Nursing." Edited by Honnor Morten.

P. S. KING AND SON.
"Report of the Royal Commission on Vaccination. A Review of the Dissentients' Statement." By John C. McVail, M.D.

SAXON AND CO.

"Silver Thorns." By Florence H. Davies.

JOHN WRIGHT AND CO.

"Surgical Technics in Hospital Practice." By K. W. Monsarrat, M.B., F.R.C.S.E.

"An Atlas of Histology." By Arthur Clarkson, M.B., C.M.

J. B. LIPPINCOTT CO.

"Mammalian Anatomy." Part I., "The Skeleton of the Cat." By Horace Jayne, M.D., Ph.D.

BAILLIÈRE, TINDALL, AND COX.

"Lectures on the Theory and Practice of Vaccination." By Robert Cory, M.A., M.D., F.R.C.P.

### SCRAPS AND GLEANINGS.

The number of patients sent through the Cotton Districts Convalescent Fund to the various convalescent hospitals in 1897 was 2,747, the cost being £4,853, of which the patients had contributed £1,818.

The report of the Newcastle Hospital for Sick Children shows that there has been a considerable increase in every department during 1897.

At the new cottage hospital which was erected at Tavistock in 1896, 111 patients were admitted last year, an increase of 31. 508 outpatients were attended to.

Slightly fewer in-patients were attended at the Glasgow Royal Hospital for Sick Children in 1897 than in 1896. being 691 against 706. The average duration of stay has increased to 35 9 days, an advance of about six days. 6,402 cases were treated at the dispensary.

A workroom has been fitted up in the Birmingham Dental Hospital where the students may have practical instruction in the process of making artificial dentures, in order to comply with the new regulations of the College of Surgeons.

The Lord Mayor of Birmingham, speaking at a meeting of the Birmingham Hospital Sunday Fund, said that the report contained one reference which was decidedly unsatisfactory—namely, that a practice was in danger of growing up of applying subscriptions to the purchase of tickets. He could sympathise with a minister in a poor district who wanted some benefit in the form of tickets for his parishioners, but it was unfair to the charities which opened their doors without tickets to deprive them of a share in that minister's collection.

Seventy-one patients were under treatment at the Tetbury Cottage Hospital during 1897.

The Gloucester General Infirmary is fast approximating to a free hospital. In 1897 only about 9 per cent. of the total number of cases admitted came into the infirmary by recommendation from subscribers.

The new fever block at the Dumfries and Galloway Royal Infirmary is now in occupation.

The income for 1897 at the Dover Hospital was £1,540, or £14 less than the expenditure.

At the Dewsbury District Infirmary in 1897 433 in, 3,115 out, and 808 home patients were treated. In addition 1,363 casualty and 452 dental cases were attended to.

The board of the Chester General Infirmary, in again reporting a decline in the subscription list, state that it is found that the subscriptions received from the Welsh Border counties are disproportionate to the large number of patients sent in from those neighbourhoods.

